# Image‐based evaluation of a commercial AI synthetic CT generator for brain and prostate MR‐only radiotherapy

**DOI:** 10.1002/acm2.70725

**Published:** 2026-08-03

**Authors:** Morgan Aire, Carson Matthews, Joshua Jones, David Duong, Christopher Schneider, Sotirios Stathakis, Olivia Shay, Krystal Kirby

**Affiliations:** ^1^ Department of Physics and Astronomy Louisiana State University Baton Rouge Louisiana USA; ^2^ Department of Radiation Oncology Mary Bird Perkins Cancer Center Baton Rouge Louisiana USA

**Keywords:** Adaptive radiotherapy, artificial intelligence, brain, image‐based evaluation, mage-guided radiotherapy, MR‐only, prostate, synthetic CT

## Abstract

**Background:**

Synthetic CTs (sCT) were developed to provide electron density information for MR‐only treatment planning. Some AI‐based sCT generators have received FDA‐clearance and better capture tissue heterogeneity through voxel‐wise mappings to improve image guidance and dose calculation.

**Purpose:**

To quantitatively characterize the image quality and geometric surrogate agreement of an FDA 510(k) cleared AI‐based synthetic CT generator (TheraPanacea) for brain and prostate MR‐only radiotherapy, using per‐structure volumetric analysis, Hounsfield unit (HU) assessment, and multi‐metric geometric evaluation.

**Methods:**

Paired CT/MR datasets from 20 brain and 20 prostate patients were retrospectively analyzed. sCTs were generated from T1w (brain) and T2w (pelvis) MR images acquired on the same day with identical immobilization. Auto‐contours from TheraPanacea and MIM Contour ProtégéAI were compared against MIM Contour ProtégéAI contours on the reference CT. Bone was segmented via a fixed‐threshold method. Evaluation metrics included contour volumes, cross‐contour HU comparisons, centroid offsets, overlap and surface‐based similarity metrics, and manual bony landmark measurements.

**Results:**

Soft‐tissue mean HU differences were <50 HU across both cohorts. Central ROIs showed <20 HU deviations, apart from heterogeneous bone and air‐filled regions. Bony structures exhibited HU range compression in the sCT, where HU differences between the sCT and the reference CT differed by a maximum of 347 ± 14 HU in the femoral heads and 455 ± 50 HU in the sacrum. Global histograms confirmed HU range compression. Centroids shifted < 2 mm for all contours (brain A‐P predominant, pelvis non‐systematic). Boundary metrics showed MDA < 5 mm, and bony landmarks differed < 1 mm between the reference CT and the sCT. Small structures in the brain had inconsistent auto‐segmentation, displaying different volumes and low Dice/JI scores. Dental implants in the sCT produced localized MR‐like susceptibility artifacts. Within manual contoured implant ROIs, the mean HU values were underestimated by ∼9,000 HU relative to CT.

**Conclusion:**

TheraPanacea sCT demonstrated acceptable soft‐tissue HU (± 50 HU) and geometric surrogate agreement (< 2–5 mm) relative to CT, with larger errors in cortical bone, air interfaces, and implants. Site‐specific QA must verify bone HU compression, peripheral geometric agreement via landmark measures, and manually review small/high‐contrast contours (optic nerves, cochleae, rectum). Institutional image and dosimetric validation, particularly with implanted devices, remains essential prior to clinical deployment.

## 1 INTRODUCTION

Computed tomography (CT) imaging has traditionally served as the gold standard for radiation therapy treatment planning, primarily because it provides Hounsfield unit (HU) values that can be converted to electron density (ED) for accurate dose calculation.^1^ The relationship between HU, ED, and photon dose has been extensively investigated, and numerous studies have quantified how uncertainties in CT‐ED calibration or HU assignment propagate into dose calculation errors for a range of treatment sites.^2^, ^3^, ^4^ However, CT imaging offers limited soft‐tissue contrast, which can affect precise tumor delineation and organ‐at‐risk (OAR) identification. Magnetic resonance (MR) imaging has increasingly been integrated into radiation therapy workflows because it provides superior soft‐tissue contrast, improving visualization of target volumes and adjacent critical structures. As a result, it may enable reduced margins and better OAR sparing, particularly in brain, abdominal, and prostate treatments.^5^, ^6^, ^7^, ^8^

In response to this enhanced anatomical detail, MR‐only treatment planning was developed to directly derive a treatment plan from MR images without a separate CT acquisition. This method has gained momentum, particularly with the clinical deployment of MR‐linear accelerators (MR‐linacs).^9^, ^10^ MR‐only workflows enable image acquisition, plan adaptation, and treatment delivery without moving the patient between imaging and treatment systems, but they lack direct ED information, which remains essential for accurate dose calculations.

To overcome this limitation, synthetic CTs have been proposed to infer ED from MR images.^11^, ^12^, ^13^, ^14^ These images can be produced using three main approaches: bulk density override (BDO), atlas‐based methods, and artificial‐intelligence (AI)‐driven models.^15^, ^16^, ^17^ BDO, used clinically in some MR‐linac implementations, assigns uniform ED values to predefined regions of interest based on prior CT data and has shown acceptable performance in relatively homogeneous anatomical regions. It can be inaccurate in heterogeneous tissues where local density variations are important for dose variations.^9^, ^18^, ^19^ Atlas‐based and AI‐based sCT techniques aim to better capture tissue heterogeneity by learning voxel‐wise mappings from MR intensity and context to HU, thereby improving both dose calculation and image guidance.^15^, ^20^

Several groups have proposed and evaluated clinical commissioning tests for AI‐based sCT generators, typically combining image‐based comparisons with dose recalculation and gamma analysis.^21^, ^22^, ^23^ In MR‐only workflows, studies have linked sCT HU errors to resulting dose differences and generally reported that HU deviations on the order of tens of HU yield dose discrepancies within 1–3% for most brain, head‐and‐neck, and pelvic plans, with high gamma pass rates and minimal DVH shifts.^21^, ^22^, ^24^, ^25^, ^26^, ^27^, ^28^, ^29^, ^30^, ^31^ Notably, Autret, et al. compared four synthetic CT generators, including the TheraPanacea system evaluated in this study, across brain and prostate MR‐only workflows, reporting HU differences and dosimetric agreement on a relatively large cohort and concluding that all four generators achieved clinically acceptable performance.^32^

While these studies provide valuable dosimetric validation, their image‐based assessments have generally been limited to measuring HU metrics and overlap/surface similarity measures for select structures.^13^, ^24^, ^25^, ^26^, ^27^, ^28^, ^33^ Such approaches establish that typical sCT errors remain clinically tolerable but provide limited insight into structure‐specific discrepancies between commercial sCTs and traditional reference CTs which may contribute to dose differences.^25^, ^33^, ^34^

The purpose of this study is to provide an in‐depth, image‐based evaluation of an FDA 510(k) cleared AI‐generated sCT algorithm (TheraPanacea, Paris, France) for brain and prostate MR‐only radiotherapy. Volumetric agreement, HU accuracy, and geometric surrogate agreement are measured between sCT and reference CT using multiple complementary metrics, and vendor‐generated contours are compared with a clinically deployed AI contouring solution.

In a primary MR‐only workflow, clinically used contours are generally delineated on the planning MR while the sCT provides electron density for dose calculation. Structures are not strictly required on the sCT itself. Contours are evaluated on the sCT as a geometric surrogate for commissioning and QA to test whether sCT‐derived anatomy remains spatially and volumetrically consistent with the reference CT. They are also used to benchmark the MR‐derived TheraPanacea contours against a clinically deployed contouring standard.

By focusing on image and geometric endpoints rather than dose, this work supplies technical performance benchmarks and a methodological framework that can be combined with dosimetric assessments with prior and future studies for comprehensive clinical commissioning of sCT generators and understanding of failure modes in MR‐only workflows.

## 2 MATERIALS AND METHODOLOGY

### 2.1 Patient population

Retrospective analysis of patient imaging data was approved by the institutional review board (IRB). This study included anonymized datasets from 53 patients who underwent both CT and MR imaging on the same day as part of standard‐of‐care radiation therapy treatment. Specifically, 25 patients who underwent brain cancer treatment and 28 patients who underwent prostate cancer treatment were initially included. Of these, five brain patients and two prostate patients were subsequently excluded due to failures in project generation by TheraPanacea's (TP) ART‐Plan platform. In every case, the failure was traced to minor DICOM header inconsistencies rather than to image quality, anatomy, or the sCT algorithm itself. For example, image‐orientation direction cosines round marginally from zero (e.g., 0.00000001 rather than 0.0). Because the generation‐failure rate is a component of clinical system performance, it was reported explicitly, though the rate reflects DICOM export/handling compatibility rather than a limitation of the sCT generation.

Six prostate patients were excluded from quantitative analysis due to the presence of brachytherapy seed implants in the prostate. The exclusion was location‐based rather than safety‐based. Unlike dental implants in the brain cohort, which lie outside analyzed soft‐tissue OARs, prostate seeds lie within the prostate target and adjacent analyzed pelvic structures, where they may introduce large, localized HU outliers into the target. However, patients with prostate seeds were included in qualitative implant analysis. The final cohort thus consisted of twenty brain (mean age: 59.9 years; mean weight: 77.7 kg.; range: 45–113 kg.) and twenty prostate cancer patients (mean age: 70.7 years; mean weight: 93.0 kg.; range: 68–115 kg.).

### 2.2 Image acquisition

CT scans were acquired using a dedicated radiation therapy CT simulator (GE Discovery RT 590, GE Healthcare, Chicago, IL, USA) institutional planning protocols. All CT scans were acquired in extended‐HU mode with an in‐plane voxel resolution of 0.98 mm x 0.98 mm and reconstructed with the standard kernel. No metal artifact reduction settings were used. Brain CT images were acquired with a slice thickness of 1.25 mm at 140 kVp, and pelvic CT images were acquired at 2.5 mm slice thickness at 120 kVp. MR imaging was performed on a 1.5 T MR simulator (MAGNETOM Sola, Siemens Healthineers, Erlangen, Germany). CT and MR scans were acquired sequentially on the same day, using the same immobilization devices, to minimize anatomical variation and setup differences between modalities.

For sCT generation, TheraPanacea requires a T2‐weighted (T2w) sequence for prostate patients and a T1‐weighted (T1w) sequence for brain patients. Specific vendor‐recommended sequence parameters beyond sequence type are not publicly available. Routine clinical imaging protocols were used, with pulse sequence parameters summarized in Table 1. For all scans, a 3D distortion correction was applied, and B0 distortion correction was not applied, per institutional clinical practice. Brain imaging extended anatomically from the superior cranial surface to the C2 vertebra, while prostate imaging spanned from the perineum to the S3 vertebra.

**TABLE 1:** MR sequence parameters for pelvis and brain synthetic‐CT generation.

Parameter	Pelvis	Brain
Scan	T2 SPACE	T1 MPRAGE
In‐plane resolution	1.5 × 1.5 mm^2^	1.0 × 1.0 mm^2^
Base matrix size	256 × 256	256 × 256
Slice thickness	1.56 mm	1.00 mm
Slice oversampling	100%	33%
Flip angle	150°	8°
Repetition time (TR)	1500 ms	2200 ms
Echo time (TE)	140 ms	1.8 ms
Number of averages	2	2
Acquisition direction	Axial	Axial

For brain patients, gadolinium‐based contrast agents were administered for the T1w scans used in this study, in accordance with institutional practice for initial target and OAR delineation, as contrast enhancement improves visualization of brain lesions and surrounding critical structures.^35^, ^36^, ^37^ TheraPanacea does not currently provide explicit recommendations regarding contrast use for sCT generation. Therefore, post‐contrast T1w images were used as input for this cohort.

### 2.3 Synthetic CT and contour generation

The TheraPanacea sCT generator (MR‐Box in ART‐Plan) employs an end‐to‐end ensemble of self‐supervised GANs incorporating cycle consistency and was trained on weakly aligned pelvic CT‐MRI pairs, as described in vendor‐collaborative work by Autret, et al.^32^ The network learns to map MR images to corresponding CT‐like HU values while enforcing anatomical consistency through cycle‐consistency losses. For subsequent dose calculations in our treatment planning system, the same scanner‑specific CT‑to‑electron‑density (CT‑ED) calibration curve is applied to both planning CT and sCT images. This CT‑ED curve was commissioned and, where necessary, adjusted to match our CT simulator's HU–ED calibration prior to clinical use. Detailed implementation and training parameters are proprietary to the vendor. The description here is based on published methodology.

MR image sets were exported to ART‐Plan for sCT generation. The resulting sCT and reference CT images were rigidly registered in MIM (MIM Software, Cleveland, OH, USA), and contours were transferred and cropped to exclude regions outside the shared field of view.

Two automated contour sets were created for the sCT. The first consisted of TheraPanacea‐generated contours derived from the initial MR images and propagated to the sCT. The second contour set was generated using the clinically deployed AI contouring solution, Contour ProtégéAI (CPAI) (MIM Software, Cleveland, OH, USA) on both the reference CT and sCT. Two comparisons were performed. The first compared the two images using the same contouring algorithm, CPAI, on both. The second comparison used the two contour sets on the sCT to compare the accuracy of the TheraPanacea contours to the clinical standard. Because the TheraPanacea contours are generated on the MR and propagated to the sCT rather than directly drawn on the sCT, these comparisons isolate distinct effects. Holding the algorithm constant (CPAI on CT vs. sCT) isolates image‐related differences, whereas holding the image constant on the sCT (TheraPanacea vs. CPAI) isolates differences attributable to the contouring approach and benchmarks the MR‐derived contours against the clinically deployed standard.

Bone contours were generated on both the sCT and reference CT using fixed‐threshold segmentation (lower limit: 147 HU^38^, ^39^), as neither software offered automated global bone contouring methods beyond the femoral heads and sacrum.

Only anatomical contours common to both contouring systems were included in the analysis. For brain patients, these regions included the brain, cochleae, lenses, lacrimal glands, optic chiasm, optic nerves, and spinal cord. For prostate patients, analyzed structures included the femoral heads, penile bulb, prostate, rectum, and sacrum, in addition to global bone contours.

### 2.4 Analysis

All quantitative analysis was performed in MIM unless otherwise specified. Image agreement between sCT and reference CT was evaluated using contour volume, HU statistics, and geometric surrogate metrics. The Wilcoxon signed‐rank test was used to assess paired differences in volume, HU, and geometric agreement. Statistical significance thresholds were set at *p* = 0.05, *p* = 0.01, and *p* = 0.001.

Perplexity AI (Perplexity AI, Inc., San Francisco, CA, USA) was used to assist with manuscript editing and the refinement of graphical R code for figure generation.

#### 2.4.1 Contour volume evaluation

Contour quality was assessed by comparing structure volumes between sCT and reference CT and by qualitative inspection of contour alignment with anatomical boundaries. Volumes from the CPAI contours on the sCT and reference CT were compared, and the two sCT contour sets were compared to identify systematic volume biases and structure‐dependent discrepancies.

#### 2.4.2 Hounsfield unit comparison

HU accuracy of the AI‐generated sCT was evaluated with four complementary strategies, each designed to probe different sources of error.

1. Cross‐contour comparison

For each structure, mean, minimum, and maximum HU values were extracted from each contour set and compared with the corresponding CPAI reference CT contours. This approach leverages clinically relevant structure sets to assess HU variability over entire organs or bony regions.

2. Central region‐of‐interest (ROI) analysis

To minimize partial‐volume and heterogeneity effects, a spherical ROI (diameter = 10 mm) was placed at the geometric center of each evaluated structure, ensuring the sphere was fully contained within the original contour in both CTs. ROIs were propagated to the sCT through rigid registration, and mean HU values were recorded on both datasets. This analysis isolates HU consistency within the most homogeneous portion of each organ or bony landmark. This comparison was performed on a subsection of 9 brain and 8 pelvic scans.

3. Deformed CT‐to‐sCT contours

Reference CT contours were deformably registered onto the sCT images. By standardizing a single contour set across both modalities, this method removes segmentation bias and enables direct voxel‐wise comparison of mean, minimum, and maximum HU values within identical volumes. This comparison was performed on a subsection of 9 brain and 8 pelvic scans.

4. Histogram comparison

A HU histogram of the external contours between the reference CT and the sCT was generated to further evaluate possible discrepancies between HU ranges. One representative example for each of the anatomical regions is provided.

#### 2.4.3 Geometric analysis

To find potential spatial discrepancies in vivo, a multi‐tiered set of geometric surrogate metrics was applied, focusing on predominantly rigid bony anatomy where intra‐session anatomical variability is minimal.

1. Rigid co‐registration and centroid analysis

After rigid registration of sCT to reference CT, paired sCT and CT contour centroids were computed in the right‐left (Δ x), anterior‐posterior (Δ y), and superior‐inferior (Δ z) directions. Translational offsets and resultant Euclidean displacements were recorded. Consistent directional offsets may indicate systematic geometric differences between modalities.

2. Volumetric and boundary‐based similarity metrics

Following rigid alignment, corresponding contours were evaluated with four agreement metrics to characterize both overlap and boundary agreement:

- Dice similarity coefficient (DSC)
- Jaccard index (JI)
- Mean distance‐to‐agreement (MDA)
- 95th‐percentile Hausdorff distance (HD_95_)

These measurements are sensitive to systematic shifts, local boundary mismatches, and shape changes between sCT and CT contours, and could act as indirect indicators of geometric discrepancies.

3. Anatomical linear measurements

To cross‐validate the volumetric‐ and boundary‐based metrics with direct anthropometric measures, linear dimensions of rigid bony landmarks were measured by a board‐certified medical physicist:

- Brain cohort: maximum anterior‐posterior and right‐left skull diameters on the axial plane.
- Prostate cohort: inter‐femoral‐head distance and pubic‐symphysis length on axial and coronal planes, respectively.

Mean differences between sCT and reference CT measurements were recorded. Because these landmarks are expected to remain stable between CT and MR acquisitions, systematic differences in these linear dimensions between sCT and reference CT could indicate large‐scale geometric scaling or distortion effects. These measurements were performed on a subsection of the cohort: nine brain patients and eight prostate patients.

#### 2.4.4 Qualitative analysis of implanted devices

To explore sCT behavior in the presence of high‐density implants, cases with dental hardware and prostate seed implants were qualitatively reviewed by a board‐certified medical physicist. Differences in artifact appearance between sCT and CT, and their potential impact on anatomical visualization, were documented.

## 3 RESULTS

### 3.1 Contour‐volume agreement

**FIGURE 1:**
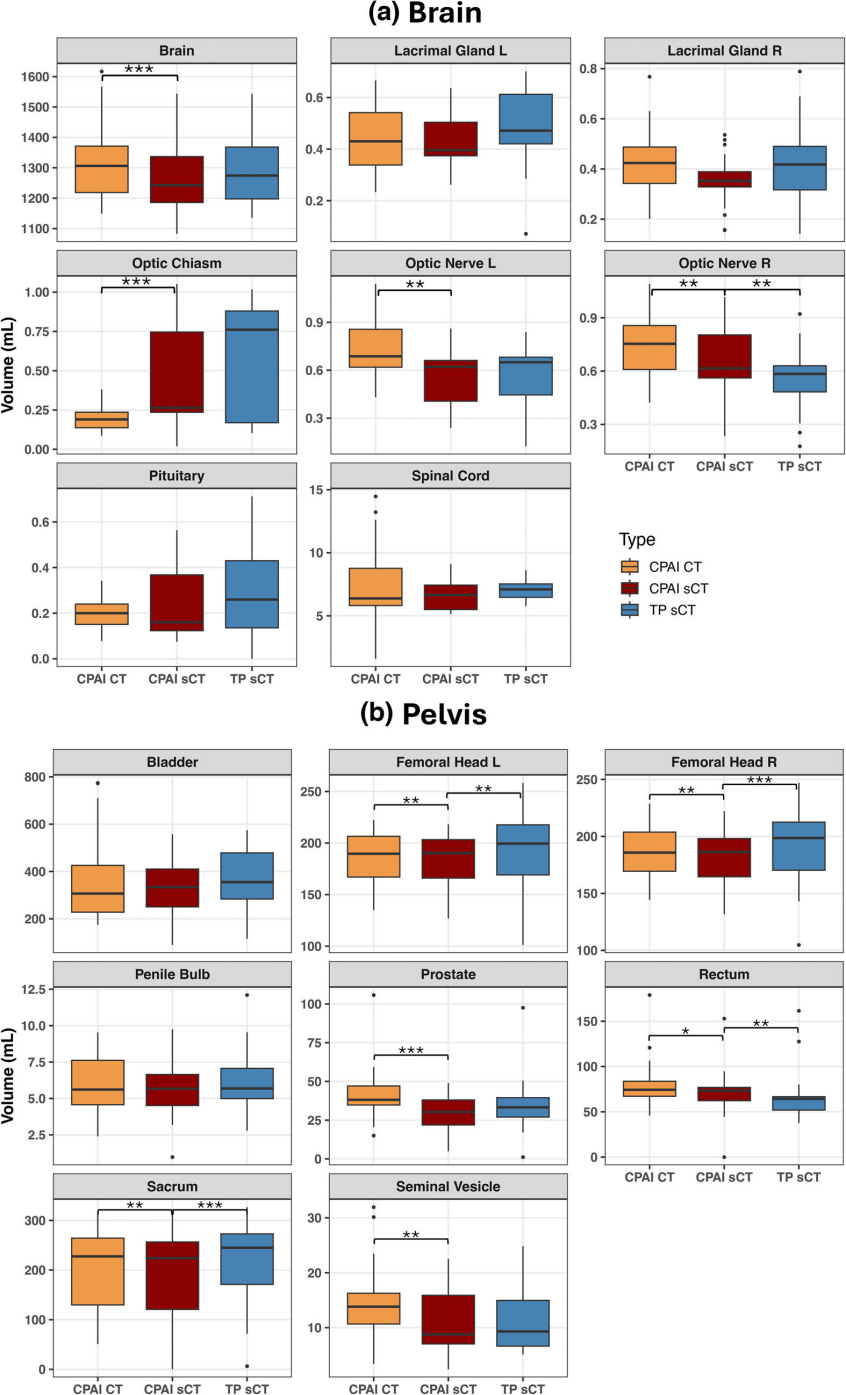
Contour volumes for the reference CT and two synthetic CT contour algorithms, Contour ProtégéAI (CPAI) and TheraPanacea (TP), for (a) brain and (b) pelvic structures. Comparisons between the CPAI CT and the CPAI sCT were performed to assess image‐based differences, and contour comparisons on the sCT between the CPAI and TP contours were evaluated for algorithmic‐based differences. (*** = p < 0.001, ** = p < 0.01, * = p < 0.05).

Volumes for brain structures on sCT and reference CT contours are summarized in Figure 1a. The CPAI sCT contouring algorithm had different volumetric measurements from the reference CT for the brain, optic chiasm, and optic nerves. Between the two algorithms on the sCT, only the right optic nerve noted a volumetric difference. Large interquartile ranges were noted, though the small size of the inner cranial structures can inflate differences. Automated detection of the cochleae and lenses was unreliable, and these structures were excluded from quantitative analysis but retained for qualitative review.

Pelvic contour volumes are shown in Figure 1b. Volumetric differences between the sCT and reference CT are noted in the femoral heads, prostate, rectum, sacrum, and seminal vesicles. Differences between the CPAI and TheraPanacea contours are noted between the femoral heads, sacrum, and rectum, where TheraPanacea‐derived femoral head and sacral volumes were consistently larger.

Qualitatively, both contouring engines captured gross anatomical boundaries reasonably well; however, jagged external body and rectal surfaces were observed in pelvic scans, particularly along the rectum and anterior abdominal wall (Figure S1). In the brain, the optic nerves and cochleae were often partially or completely missed by at least one auto‐segmentation method.

### 3.2 Hounsfield unit (HU) comparison

#### 3.2.1 Cross‐contour comparison

**FIGURE 2:**
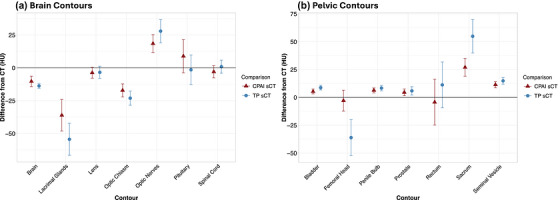
Mean Hounsfield unit difference for (a) brain contours and (b) pelvic contours between the reference CT and the sCT. The red triangle points indicate the mean HU difference between the CPAI contours on the sCT with the CPAI reference CT contours, and the blue circle indicates the mean difference between the TheraPanacea (TP) contours and the CPAI reference CT contours. A positive value indicates a greater TheraPanacea sCT value than reference CT value. Error bars indicate the standard error of the mean.

Mean HU values for the brain are visualized in Figure 2a. Maximum, mean, and minimum values for each structure are summarized in Table S1. Across brain contours, the sCT differed from the CT for most minimum HU values, with the largest mean differences found in the cochleae (‐382 ± 76 HU). When comparing differences between each sCT contour and the reference contour, both sCT contour sets performed similarly, and mean HU values were within 50 HU of the reference CT, excluding the cochleae.

Pelvic cross‐contour HU differences are illustrated in Figure 2b, and maximum, mean, and minimum values are in Table S2. Maximum HU values for the prostate approached those observed in bone, reflecting inclusion of bony tissue within both sCT contour sets. This marks the need for additional review of contours and modifications when necessary. Neither algorithm should be used without revisions. The sCT exhibited truncated maximum HU values for bony structures, including the global bone (1772 ± 763 HU difference), femoral heads (‐347 ± 14 HU difference), and sacrum (455 ± 50 HU difference), relative to CT. Despite these differences in extremes, mean HU values for all pelvic sCT contours were within approximately 50 HU of the corresponding reference CT values.

#### 3.2.2 Central region‐of‐interest analysis

Mean HU differences for centrally located ROIs between sCT and CT are found in Table 2 (a brain and B. pelvis). A positive difference indicates the sCT values were greater.The largest HU deviations were observed in bones and in structures containing air pockets, whereas approximately uniform soft‐tissue regions showed the smallest differences. For the brain, most central ROIs were within about 10 HU of the reference CT on average, with the occipital bone as the main outlier. For the pelvis, all central ROIs were within about 20 HU, except for the femoral heads, which demonstrated larger HU discrepancies consistent with their heterogeneous composition.

**TABLE 2:** Hounsfield unit differences in a spherical ROI between sCT and reference CT images for A. brain and B. pelvic scans.

Panel A Differences in brain structures							
	Corpus Callosum	Pons	Occipital Bone	Optic Chiasm	Vitreous Humor	Adipose Tissue	Parotids
Mean Difference (HU)	+1.4	+7.9	+75.4	+1.4	+7	+7.9	−2.5

Panel B Differences in pelvic structures							
	Bladder	Prostate	Rectum	Seminal Vesicles	Femoral Head L	Femoral Head R	Adipose Tissue
Mean Difference (HU)	−1.6	−9.3	−12.3	−18	+42.8	+23.4	−14.9

Mean HU differences derived from deformably propagated reference‐CT contours revealed similar trends to those observed in the rigidly registered cross‐contour analysis in section 3.2.1., with relatively small mean HU differences in most soft tissues and larger discrepancies and range compression in bony and air‐containing structures. Plots of the brain and pelvis HU differences can be found in Figure S2.

**FIGURE 3:**
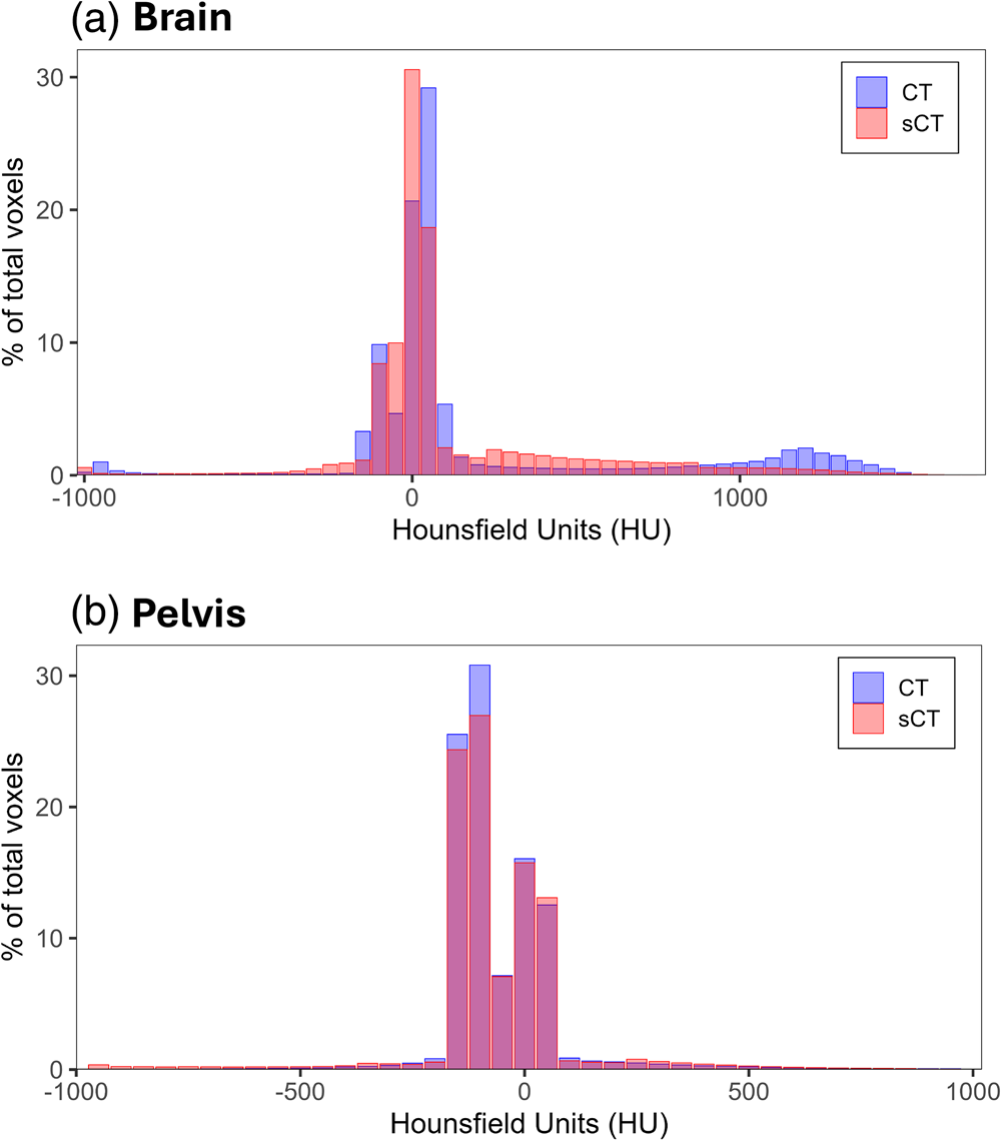
CT vs. sCT Hounsfield unit distributions for a representative (a) brain and (b) pelvis patient. A histogram displaying the distributions of CT (blue) and sCT (red) as a function of the percentage of total voxels at that value.

Representative global HU histograms are provided in Figure 3 for the (a) brain and (b) pelvis. For visualization, only histogram bins containing at least 0.1% for brain and 0.01% for pelvis of voxels are displayed. In both anatomical sites, the overall HU distributions of the sCT closely resembled those of the reference CT, with minor differences in the tails of the distributions corresponding to the observed truncation of extreme HU values in bone and air‐containing regions for cranial contours.

#### 3.2.3 Qualitative observations

**FIGURE 4:**
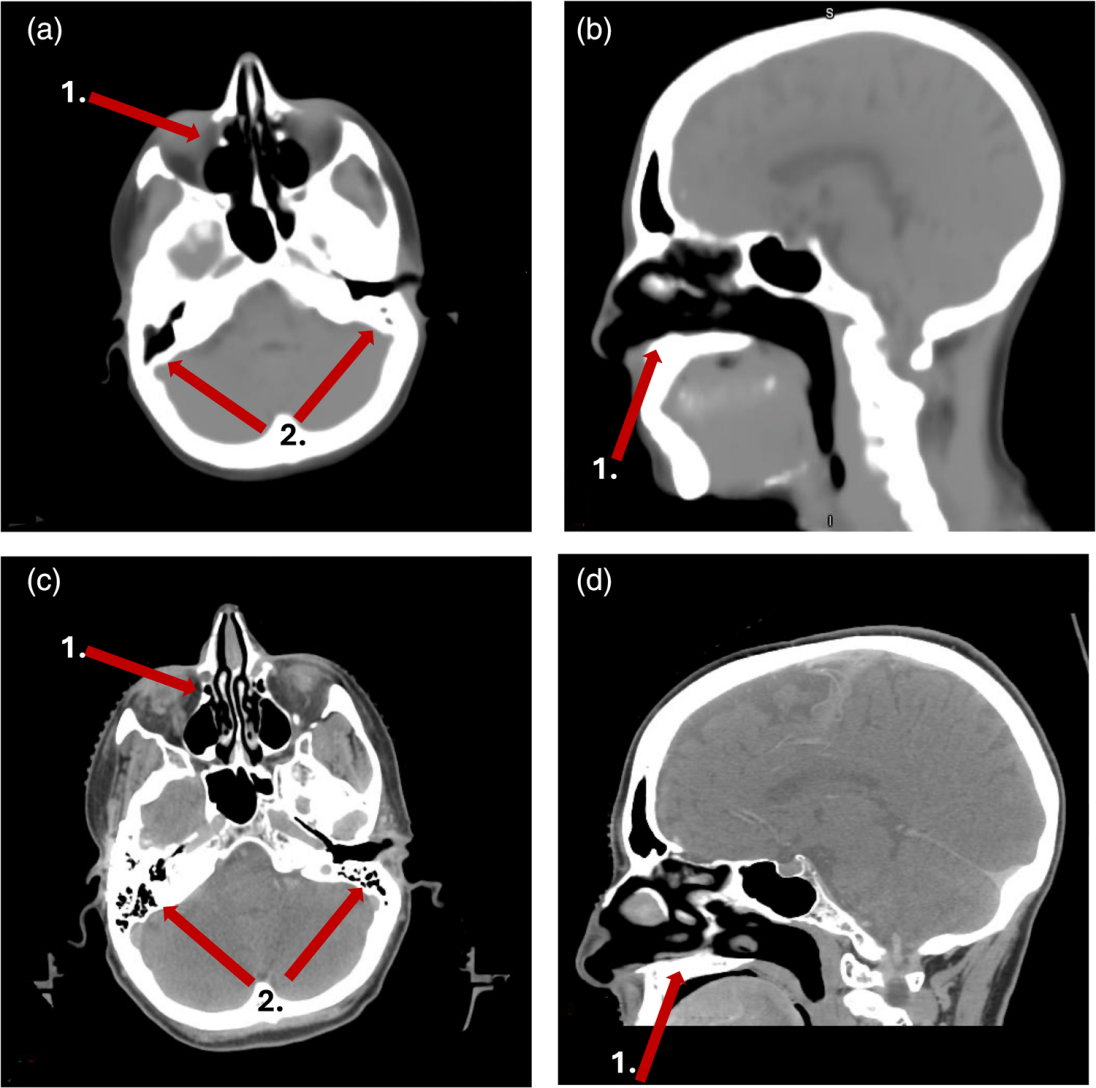
Axial and sagittal slices of synthetic CT (a, b) and reference CT (c, d) illustrating HU homogenization: nasal cavities (Arrow 1), occipital bone (Arrow 2).

Visual inspection revealed that the synthetic CT differed most from the reference CT in highly heterogeneous anatomical regions (Figure 4). Sinus cavities and spongy bone were partially smoothed together on the sCT, producing attenuation differences in complex structures such as the occipital bone, mastoid air cells, and middle ear structures. Correspondingly, ear‐related contours were frequently absent or grossly underestimated compared with the reference CT.

**FIGURE 5:**
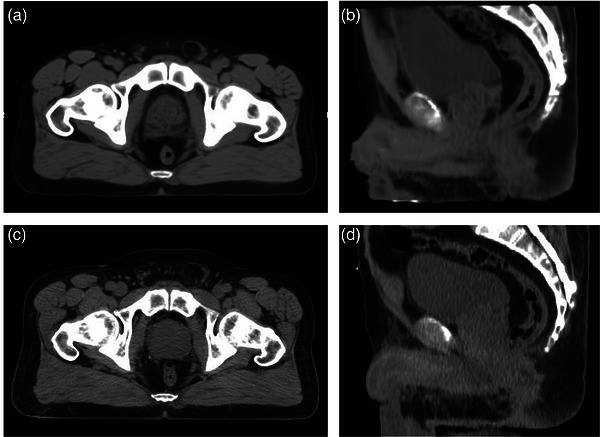
Axial and sagittal slices of synthetic CT (a, b) and reference CT illustrating slight homogenization in the femoral heads and sacrum.

The pelvic sCT images in Figure 5 showed similar patterns with regards to bone and air‐tissue smoothing, though smoothing was less pronounced. Due to the CT and the MR acquired sequentially, it is more difficult to qualitatively compare the air‐tissue boundaries as they are temporally dependent.

### 3.3 Geometric agreement assessment

#### 3.3.1 Centroid displacement

**FIGURE 6:**
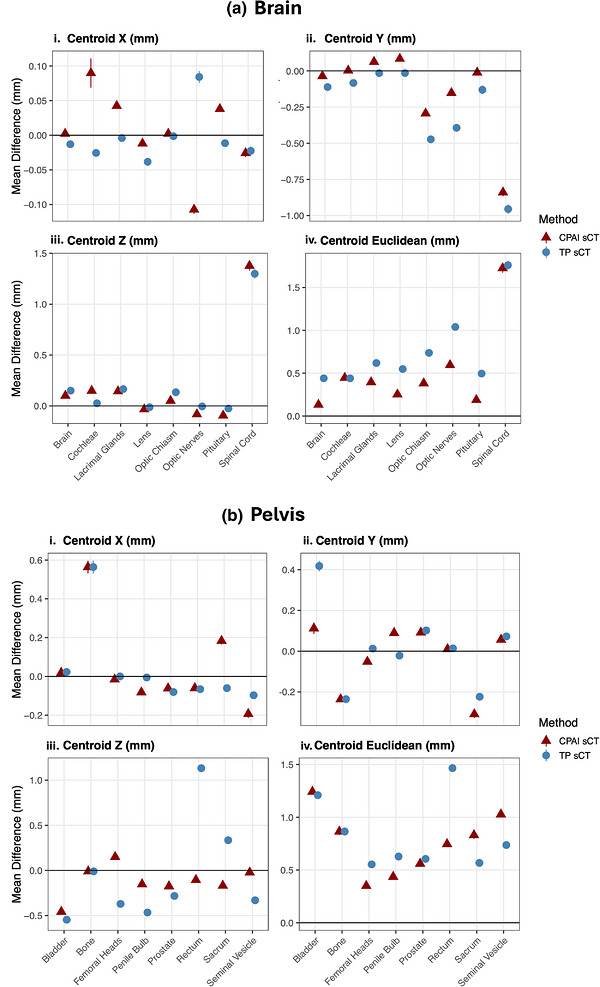
Differences in centroid location on sCT contours compared to the reference‐CT for (a) brain and (b) pelvic patients. Both sCT contour sets, CPAI and TheraPanacea (TP), were used for analysis. Differences were measured in the (i) x‐direction (left‐right), (ii) y‐direction (anterior‐posterior), (iii) z‐direction (superior‐inferior), and (iv) Euclidean distance. The bladder and seminal vesicle were excluded from pelvic subplot iv due to inconsistent contouring. Error bars were derived from standard error of the mean.

Figure 6 summarizes translational offsets for the (a) brain and (b) pelvis between sCT and reference‐CT contour centroids in the left‐right (Δx), anterior‐posterior (Δ y), and superior inferior (Δ z) directions, together with the resultant Euclidean offset for brain and pelvis scans, respectively.

For the brain cohort, the largest systematic offset with respect to the reference‐CT was observed along the anterior‐posterior (Δy) axis, driven predominately by TheraPanacea‐generated contours; lateral (Δx) offsets remained negligible. The spinal cord showed a consistent 1.5 mm caudal shift for both contouring systems.

In the pelvis, there was no consistent direction in systematic offset, and all centroid differences were less than 1.5 mm.

#### 3.3.2 Volumetric and boundary‐based similarity metrics

**FIGURE 7:**
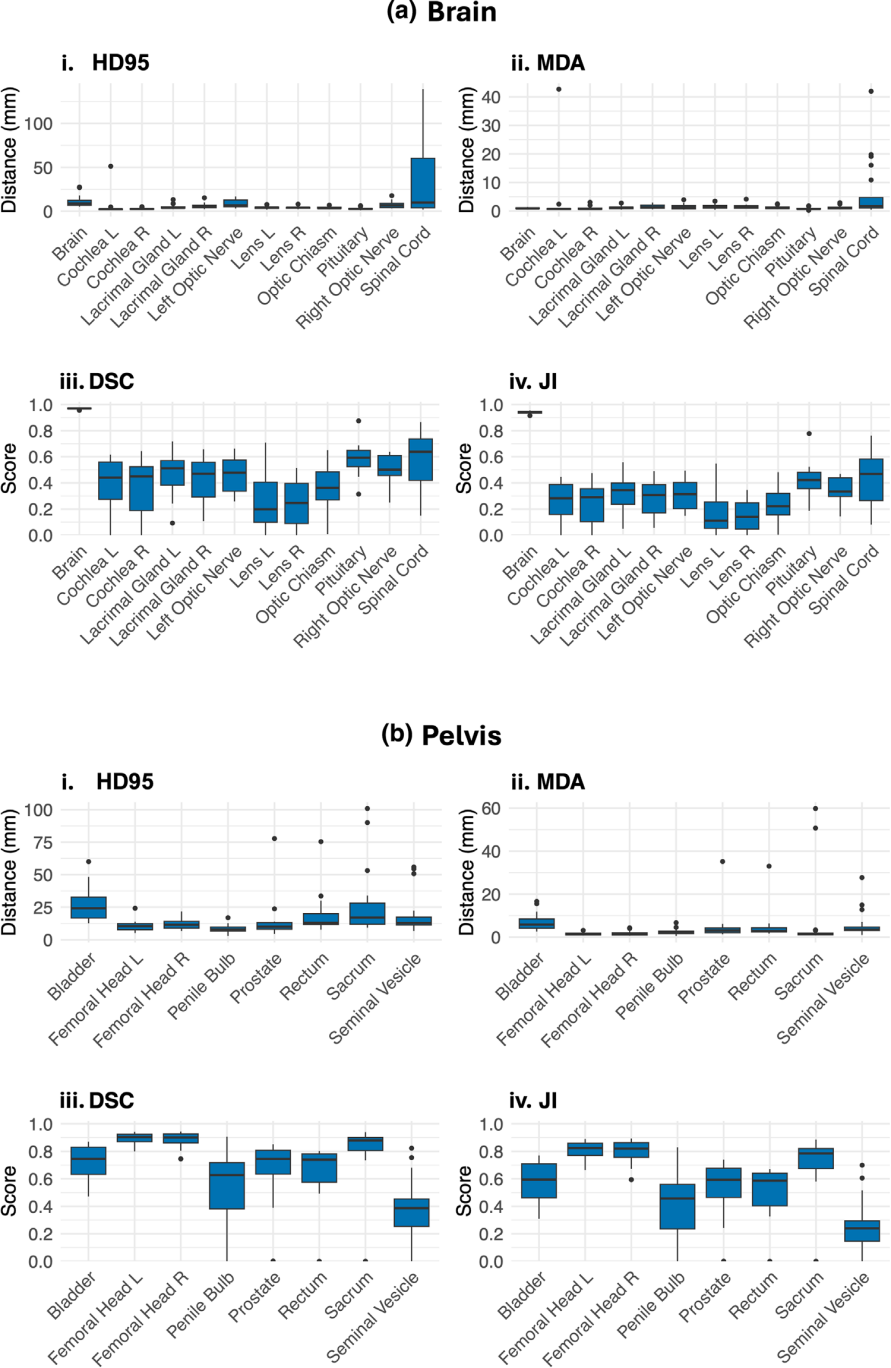
Segmentation metrics across contours and patients for (a) brain and (b) pelvic scans. The (i) Hausdorff Distance (HD95), (ii) Mean Distance to Agreement (MDA), (iii) Dice Similarity Coefficient (DSC), and (iv) Jaccard Index (JI) were calculated between CPAI‐generated contours for the sCT and the CT.

Brain and pelvis segmentation comparison metrics between sCT and the reference CT are shown in Figure 7. For the brain, the global brain contour was the only contour to achieve high Dice and Jaccard scores. All other contours had poor agreement, which is consistent with increased sensitivity of these metrics to subtle boundary variations in small volumes. Boundary‐based agreement metrics (HD95 and MDA) indicated mean distance to agreement of less than 5 mm for all structures, but the spinal cord had a large interquartile range.

In pelvic scans, the HD95 and MDA were largest in the bladder. The femoral heads and sacrum achieved moderate to high agreement for DSC and JI (scores > 0.8), and all other structures showed poor agreement, reflecting the challenge of segmenting small or deformable organs.

**FIGURE 8:**
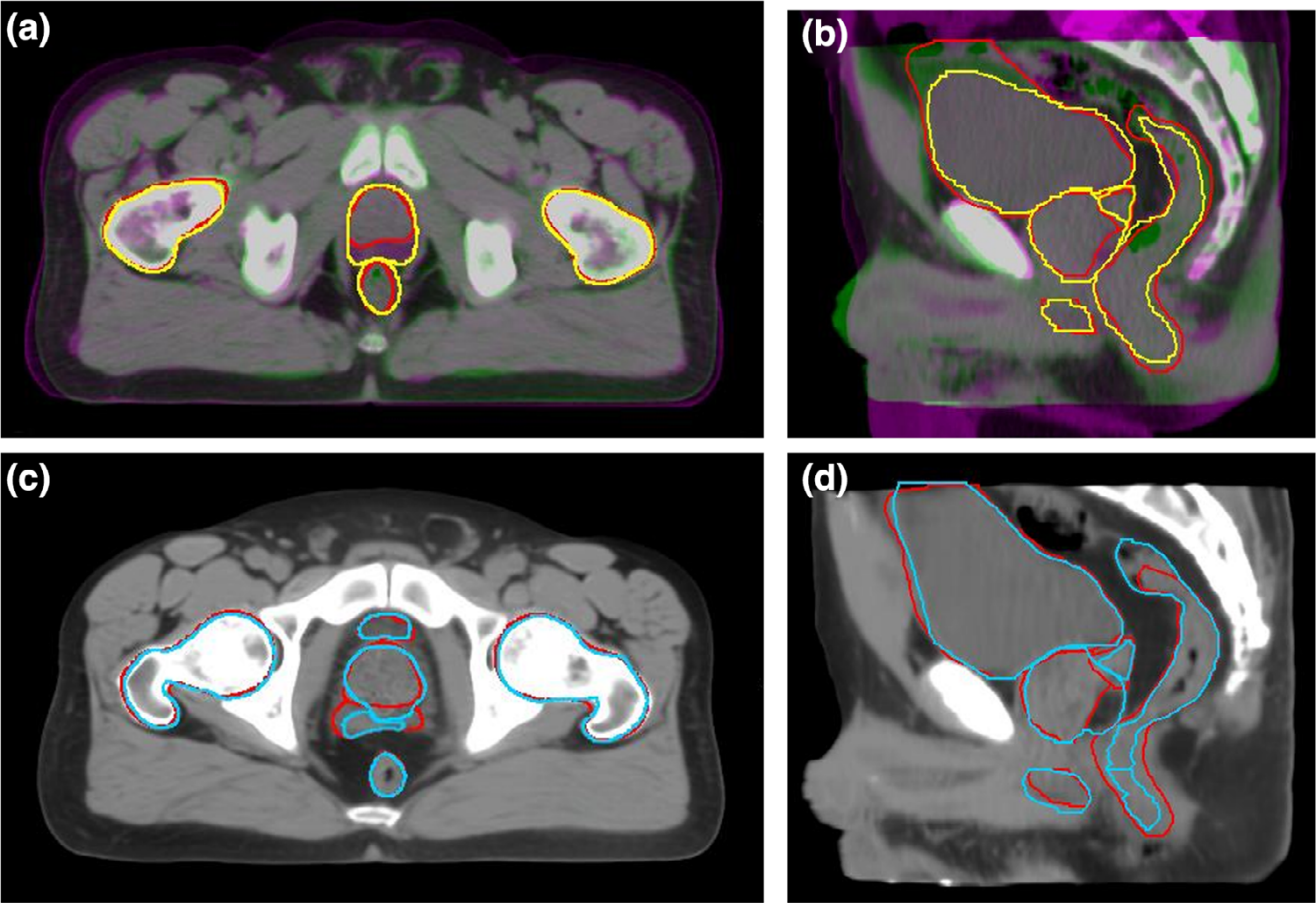
Representative pelvic contour overlays for a prostate patient, shown in axial (left) and sagittal (right column) views. Top row (a, b): CPAI contours generated on the reference CT (yellow) versus on the sCT (red), displayed on the rigidly registered CT (magenta)‐sCT (green) fusion to compare both sets on common anatomy. This corresponds to the geometric agreement quantified in Figure 6. Bottom row (c, d): CPAI (red) versus TheraPanacea (light blue) contours, both on the sCT. Structures shown: femoral heads, bladder, penile bulb, prostate, seminal vesicles, and rectum.

In the representative overlays (Figure 8a,b), the CPAI contours on the sCT and reference CT followed the same anatomical boundaries with only modest, structure‐dependent offsets, indicating that the differences largely arise from the differing input‐image rather than gross mis‐delineation. The largest visible differences occurred for the penile bulb and seminal vesicle, consistent with lower Dice and higher HD95 values.

#### 3.3.3 Manual linear measurements

**FIGURE 9:**
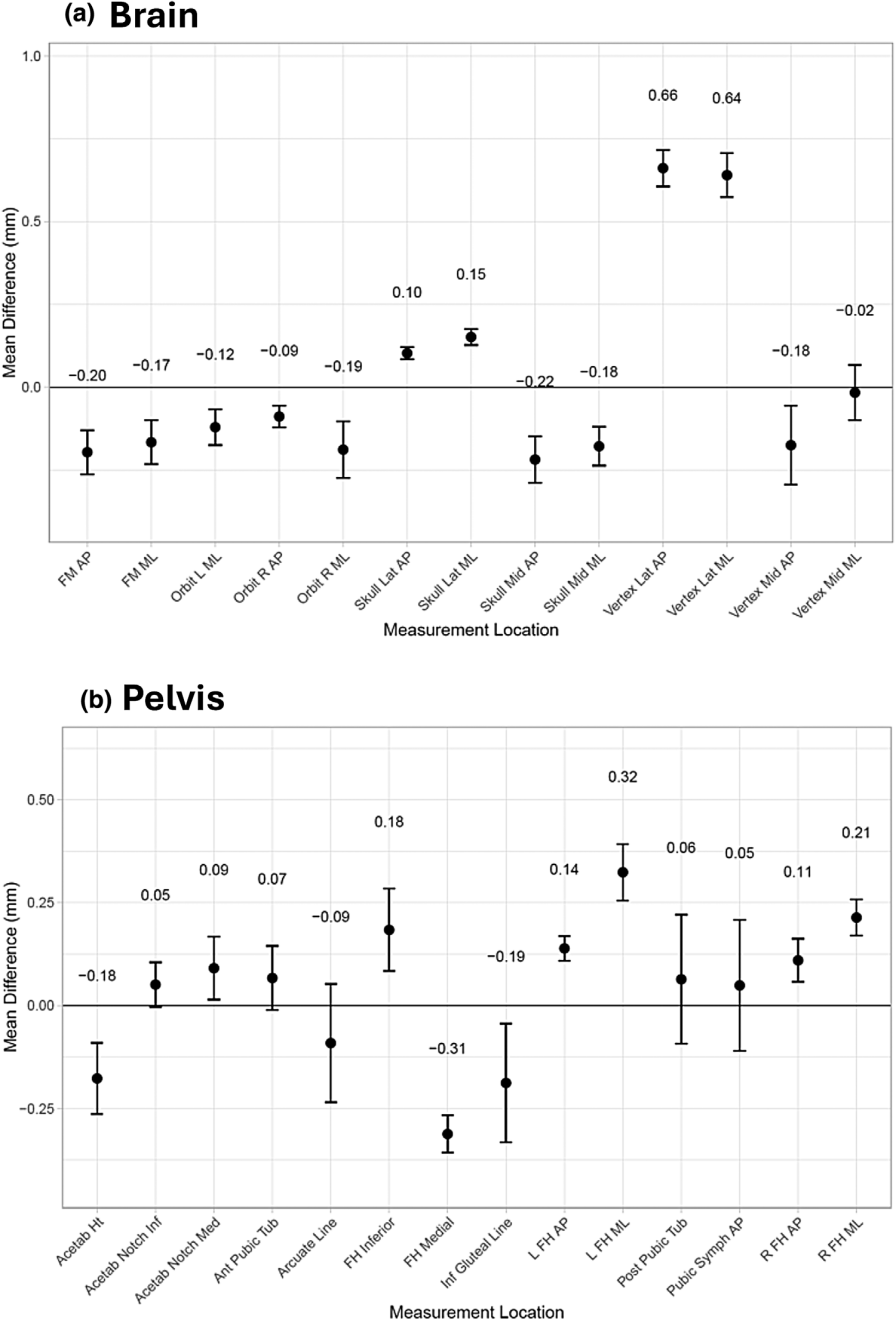
Difference measurements for (a) cranial and (b) pelvic bony landmarks. Abbreviations used: FM = Foramen Magnum, AP = Anteroposterior depth, ML = Mediolateral width, L/R = Left/Right, FH = Femoral Head, Ht = Height. A positive difference indicates a larger sCT value.

Manual skeletal measurements on matched axial or coronal slices are shown in Figure 9 for A. brain and B. pelvis and broadly agreed with the volumetric and boundary‐based agreement findings. For the brain, skull dimensions differed by up to 0.66 mm relative to the reference CT. Pelvic bony anatomy showed somewhat smaller deviations, where femoral head dimensions differed by up to 0.32 mm, and the pubic symphysis differed by up to 0.06 mm.

### 3.4 Implanted devices

**FIGURE 10:**
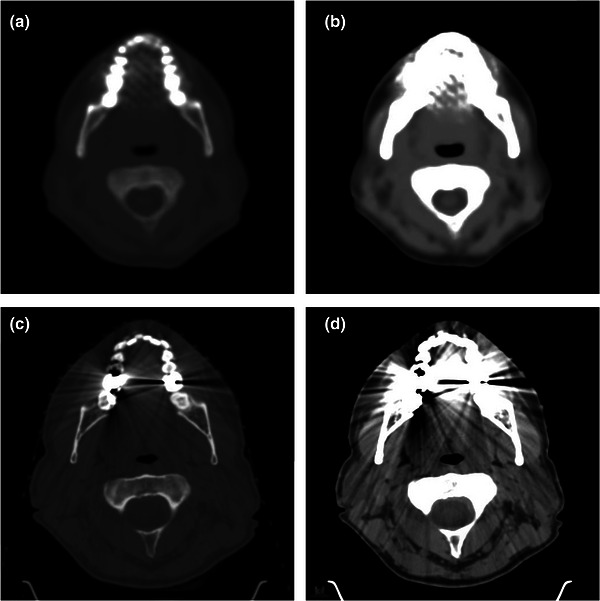
Dental implants on the synthetic‐CT in (a) bone and (b) soft‐tissue contrast windows. Implants on reference‐CT for (c) bone and (d) soft‐tissue windows. Streaking artifacts are eliminated in the sCT, and artifacts instead are more localized to the bones.

**FIGURE 11:**
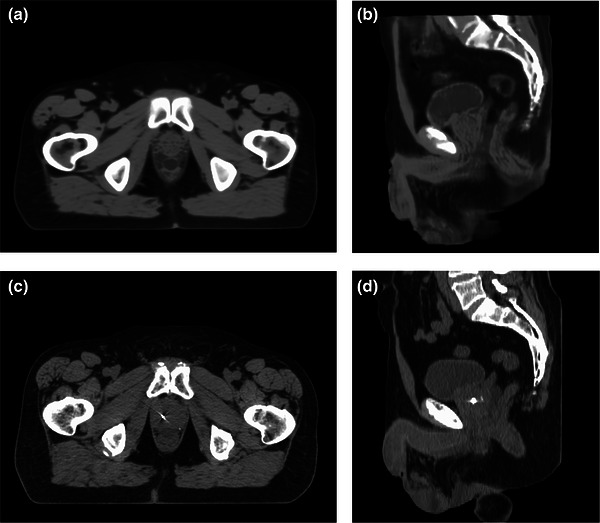
Prostate seed implants on the synthetic‐CT in (a) axial and (b) sagittal views. Implants on reference‐CT in (c) axial and (d) sagittal views. Seeds are absent in the sCT images.

Metallic implants such as dental implants (Figure 10) and prostate seeds (Figure 11) were qualitatively reviewed to assess their impact on sCT appearance and contouring. Streaking artifacts commonly observed around metal on CT were notably absent from the sCT for dental implants. Instead, reduced artifact spread into adjacent tissues was noted, with artifacts imitating MR susceptibility artifacts. In manual contours of the dental implant site, a mean HU difference of approximately 9,000 HU was noted.

Prostate seeds were notably absent from the sCT. In prostate ROI contours, a mean HU difference of 9.23 HU and a max HU difference of approximately 9,500 HU was noted.

## 4 DISCUSSION

Our results are broadly consistent with prior sCT studies that have reported modest HU and geometric errors, but they add more granular, structural‐level benchmarks that are directly applicable to commissioning and QA.

### 4.1 Contour volume evaluation

The divergence of the CPAI cranial contours and their wide volumetric ranges reflect the difficulty of consistently auto‐segmenting small structures. The optic nerves and cochleae were often missed or partially captured, inflating volumetric differences despite small absolute discrepancies. These findings demonstrate the need for manual review of small, high‐contrast OARs. The improved soft tissue delineation when using an MR for contouring rather than a CT may improve the accuracy of the TheraPanacea contours above the CPAI contours.

Although there were statistical differences between contours, particularly between the sCT and reference CT CPAI pelvic contours, the means and interquartile ranges were very similar. Whether this is a clinically meaningful difference may be more important than statistical differences. In the pelvic scans, differences in the bone volume for the femoral heads and sacral contours may be due to the less‐defined distinction between cortical and spongy bone on the sCT. The bladder and rectal volumes are expected to differ between CT‐ and MR‐based contours because of the physiologic motion and variable filling as the scans were acquired at different time points. TheraPanacea also provides multiple rectal definitions (“rectum” and “anal‑rectum”) with different inferior endpoints, and the “anal‑rectum” contour more closely resembled the CPAI definition. Given that all patients in this cohort received prostate radiotherapy, these systematic differences in target and OAR volumes highlight the importance of structure definition and clinician review when integrating sCT‑based auto‑contouring into clinical workflows.

Qualitatively, the sCT‑derived external body and rectum contours often exhibited pronounced “saw‑tooth” boundaries that were not seen in CPAI contours on either the CT or sCT. These jagged edges suggest a sensitivity of the TheraPanacea engine to low soft‑tissue contrast and bowel‑gas interfaces on the MR input, as well as potential amplification of minor image noise at tissue boundaries. In some cases, the external contour also followed faint ghosting artifacts visible only in low‑contrast window settings, leading to noticeable deviations from the true skin surface. For commissioning and routine use, additional contour smoothing or manual editing of the external body contour may be advisable to ensure accurate patient outlines for planning and dose calculation.

### 4.2 Hounsfield unit evaluation

The HU differences were within 50 HU for both the rigidly registered contours and the deformed soft‐tissue contours, implying volume‐wise consistency.

The bone HU range compression likely reflects anatomical heterogeneity, partial‐volume effects, and algorithmic smoothing, with the extreme values disproportionately set by a small fraction of voxels. Excluding the rarest (< 0.1%) brings the pelvic external‐body histograms into close agreement, while heterogeneous cranial regions remain divergent (Figure 3).

From a dosimetric standpoint, the observed range compression in bone may be of limited consequence for typical non‐bony targets. Photon dose downstream of a bony structure depends primarily on the integrated attenuation along the beam path, so preservation of the mean HU, as observed here, may limit downstream dose error even when extreme HU values are truncated. This hypothesis is consistent with prior reports of clinically acceptable dose agreement despite modest sCT HU errors. The compression is more likely to matter where bone or a bone‐air interface is itself near the target or where high‐Z heterogeneity dominates the local dose, which should be flagged for site‐specific verification.

The central‐ROI pattern, with large bone and small soft‐tissue deviations, is consistent with the sCT averaging trabecular and cortical components. This may be the mechanism behind the bone compression.

It is also important to note the dosimetric relevance of a given HU discrepancy depends not only on its magnitude but also on the size of the affected volume and its position along the beam path. A small mean‐HU offset distributed over a large, beam‐traversed volume (e.g., the whole brain) contributes more to cumulative attenuation than a comparable or larger offset confined to a small structure such as a lacrimal gland or lens. The structure‐specific HU differences reported here should therefore be weighted by anatomical context when prioritizing review.

### 4.3 Geometric agreement evaluation

The small anterior‐posterior centroid bias in the brain, driven mainly by TheraPanacea contours, may arise from those contours being generated on the MR rather than the sCT. Its absence in the pelvis, and the lack of any consistent directional bias across cohorts, points to registration and contouring variability rather than scanner‐related distortion as the source of these sub‐2 mm shifts.

The low Dice/Jaccard scores for small structures alongside their low MDA values show that the overlap metrics overstate disagreement. Even when boundaries stay close, the small volume perturbations collapse volumetric overlap. The spinal cord (brain) and bladder (pelvis) were the primary exceptions.

Manual linear measurements found skeletal distances differed by less than 1 mm for both the skull and pelvis. As this is the only metric not dependent on contours and extended to the edges of the field of view, the small differences do not indicate large systematic geometric uncertainties. None of these geometric surrogates isolated MR‑related distortion as the sole cause of disagreement. Instead, the combined patterns are more consistent with residual registration error, patient positioning differences, and contour variability. Further work, including dedicated phantom measurements and vendor‑specific characterization, is needed to fully understand how TheraPanacea addresses geometric distortion, and the use of scanner distortion‑correction during MR acquisition remains essential to minimize spatial error. Care should be taken when generating sCTs from diagnostic scans, where standards for geometric distortion are less strict compared with therapeutic scans.

### 4.4 Implanted devices

Metallic artifacts on the sCT differed in appearance from those on CT for dental implants, presenting as localized intensity changes and shape distortions near implants rather than the high‑intensity streaks and photon‑starvation bands typically seen on CT. This qualitative behavior may facilitate more reliable ROI delineation adjacent to metallic hardware, although this was not quantitatively assessed. By contrast, the absence of prostate seed implants on sCT may be due to their appearance as small susceptibility signal voids on a T2w scan, which TheraPanacea may map to normal tissue.

The dosimetric consequences for dental implants, given the ∼9,000 HU mean underestimation in the ROI, are unknown and could be substantial depending on beam geometry. The marked truncation of maximum HU values observed in the sCT, particularly in the skull, may be driven in part by dental implants. The large differences in HU values remain consistent with the previous hypothesis that TheraPanacea is not trained on extended HU scans.

The minimal mean HU difference in the prostate may imply minor dosimetric difference overall despite the large peak HU difference because the seed volume is small relative to the prostate.

### 4.5 Practical implications

From a practical standpoint, the present results suggest several concrete checks that can be incorporated into site‑specific commissioning and QA of this sCT solution. First, mean HU values for large soft‑tissue structures in brain and pelvis should fall within approximately ± 50 HU of the reference CT, with bone‑containing structures showing larger but still predictable biases and range compression.

Second, the extent of HU truncation for skeletal and implanted structures should be assessed on an institutional basis. A site‐specific dosimetric analysis is recommended before implementing MR‐only treatment planning for patients with implanted devices.

Third, centroid offsets and MDA values for rigid bony landmarks should generally remain within a few millimeters, and manual linear measurements can be used to confirm that skull and pelvic dimensions differ by no more than two millimeters between sCT and CT; deviations beyond these ranges should prompt review of MR distortion‑correction and registration settings.

Finally, small, high‑contrast structures such as optic nerves, cochleae, and seminal vesicles require manual review and potential adjustment, given the lower Dice/Jaccard scores and inconsistent auto‑segmentation performance observed in this cohort.

### 4.6 Limitations

The quantitative results reported here apply to patients without treatment‐region implants and for whom sCT generation succeeded. Performance in these cases is not characterized in this study. Additionally, the investigation has several limitations that should be considered when interpreting the findings and translating them into clinical practice.

1. Single‐institution technical characterization

All data were acquired at a single institution using one CT simulator and one 1.5 T MR simulator. The results should therefore be viewed as a technical characterization of this specific implementation rather than a generalized multi‑institutional performance benchmark.

2. Single‐vendor, single‐field‐strength protocol

All MR examinations were performed on a single 1.5 T MR simulator platform with vendor‑specific distortion‑correction enabled and using institution‑specific pulse sequence parameters. Performance on other MR vendors, field strengths (e.g., 3 T), or sequence configurations may differ and requires separate verification.

3. In‐vivo geometric assessment

Geometric discrepancies were evaluated using centroid shifts, overlap/boundary metrics, and linear measurements on patient anatomy. Without dedicated phantom measurements, these in‑vivo metrics cannot fully isolate scanner‑related distortion from other contributors such as registration uncertainty, patient positioning differences, and contouring variability; as such, they should be interpreted as upper‑bound estimates of potential spatial error rather than direct measurements of MR system distortion.

4. Lack of Dosimetric Correlation

This study was intentionally limited to image‑based and geometric endpoints, with the goal of providing complementary, image‑focused criteria for commissioning and QA of a commercial sCT generator. No dose recalculation or DVH analysis was performed for this cohort. Site‑ and vendor‑specific dosimetric validation remains essential before relying solely on this system for clinical MR‑only planning and decision‑making.

### 4.7 Future work

Future work will focus on expanding the patient cohort to improve statistical power and to better capture the performance of the sCT generator across a wider spectrum of anatomies, including more cases with implants (including dental and prostate seed implants), post‑surgical cavities, and other atypical features. A larger, more heterogeneous dataset will enable more precise estimation of structure‑specific HU and geometric variability and more robust identification of rare failure modes.

Given that post‑contrast T1‑weighted MR images are often used for initial brain treatment planning, whereas non‑contrast T1w images may be used for daily MR‑only adaptive treatments on systems such as the Elekta Unity,^17^ the potential influence of gadolinium contrast on sCT generation warrants dedicated study. In this work, we did not compare sCTs derived from pre‑ versus post‑contrast T1‑weighted images, and future investigations should quantify whether contrast administration alters HU distributions or geometric surrogate agreement in ways that could impact dose calculation or image‑guided workflows, and whether harmonized contrast protocols are needed to ensure consistency between planning and adaptation.

In parallel, a logical next step is to link the image‑based metrics reported here to dosimetric endpoints by recalculating clinical plans on the sCT for a subset of patients, with particular attention to challenging scenarios such as metallic implants and highly heterogeneous or air‑containing regions. Such studies would help determine how the observed HU and geometric deviations translate into dose differences in practice and would support the development of comprehensive, site‑specific commissioning and QA guidelines for MR‑only workflows using this sCT generator.

## 5 CONCLUSION

This work provides a per‑structure image‑quality and geometric characterization of a commercial AI‑based sCT generator for brain and prostate MR‑only radiotherapy. Mean HU differences for most soft‑tissue structures were within 50 HU, whereas larger HU differences occurred in cortical bone and heterogeneous, air‑containing regions. Geometric surrogate metrics (centroid shifts, surface metrics, and linear measurements) showed small but non‑negligible spatial differences, generally within a few millimeters for rigid bony landmarks, without evidence of large‑scale directional distortion. Volumetric agreement deteriorated for small, high‑contrast structures such as the optic nerves, cochleae, and seminal vesicles, highlighting the need for manual review of these contours.

These image‐based findings provide structure‐level benchmarks that can support site‐specific commissioning and QA of this sCT machine, but they do not by themselves establish dosimetric adequacy for MR‐only treatment. Used alongside dosimetric validation, the metrics reported here can be used to: (i) verify the magnitude of bone HU compression relative to CT in brain and pelvic sites, (ii) confirm geometric agreement at the field‑of‑view periphery using simple bony‑landmark measurements, and (iii) require systematic physicist review of small‑volume and air‑interface structures, including optic nerves, cochleae, seminal vesicles, rectum, and paranasal sinuses. Additionally, until further analysis on the effect of implanted devices are demonstrated, this is only recommended for patients without implanted devices in the treatment region. Larger, multi‑institutional cohorts and dedicated dosimetric studies are still needed to link these image‑based benchmarks to dose differences and to inform broader clinical adoption guidelines.

## AUTHOR CONTRIBUTIONS

**Morgan Aire**: Data acquisition; analysis. **Carson Matthews**: Data acquisition; analysis. **David Duong**: Data acquisition; analysis. **Joshua Jones**: Data acquisition, analysis. **Morgan Aire**: Data interpretation. **Carson Matthews**: Data interpretation. **David Duong**: Data interpretation. **Joshua Jones**: Data interpretation. **Sotirios Stathakis**: Data interpretation. **Christopher Schneider**: Data interpretation. **Krystal Kirby**: Data interpretation. **Olivia Shay**: Data interpretation. **Sotirios Stathakis**: Supervision. **Christopher Schneider**: Supervision. **Krystal Kirby**: Supervision. **Morgan Aire**: Methodology development. **Carson Matthews**: Methodology development. **Krystal Kirby**: Methodology development. **Sotirios Stathakis**: Methodology development. **Christopher Schneider**: Methodology development. All authors contributed to the manuscript preparation, and final editing and review.

## CONFLICT OF INTEREST STATEMENT

The authors declare no conflicts of interest.

## ETHICAL APPROVAL STATEMENT

Retrospective analysis of patient imaging data was approved by the institutional review board (IRB) under protocol IRBAM‐25‐0232.

## Supporting information



Supporting Information
