# Supplementary material for: Image‐based evaluation of a commercial AI synthetic CT generator for brain and prostate MR‐only radiotherapy
Source: J Appl Clin Med Phys. 2026 Aug 3;27(8):e70725. doi: 10.1002/acm2.70725 (PMC13433487; doi:10.1002/acm2.70725)

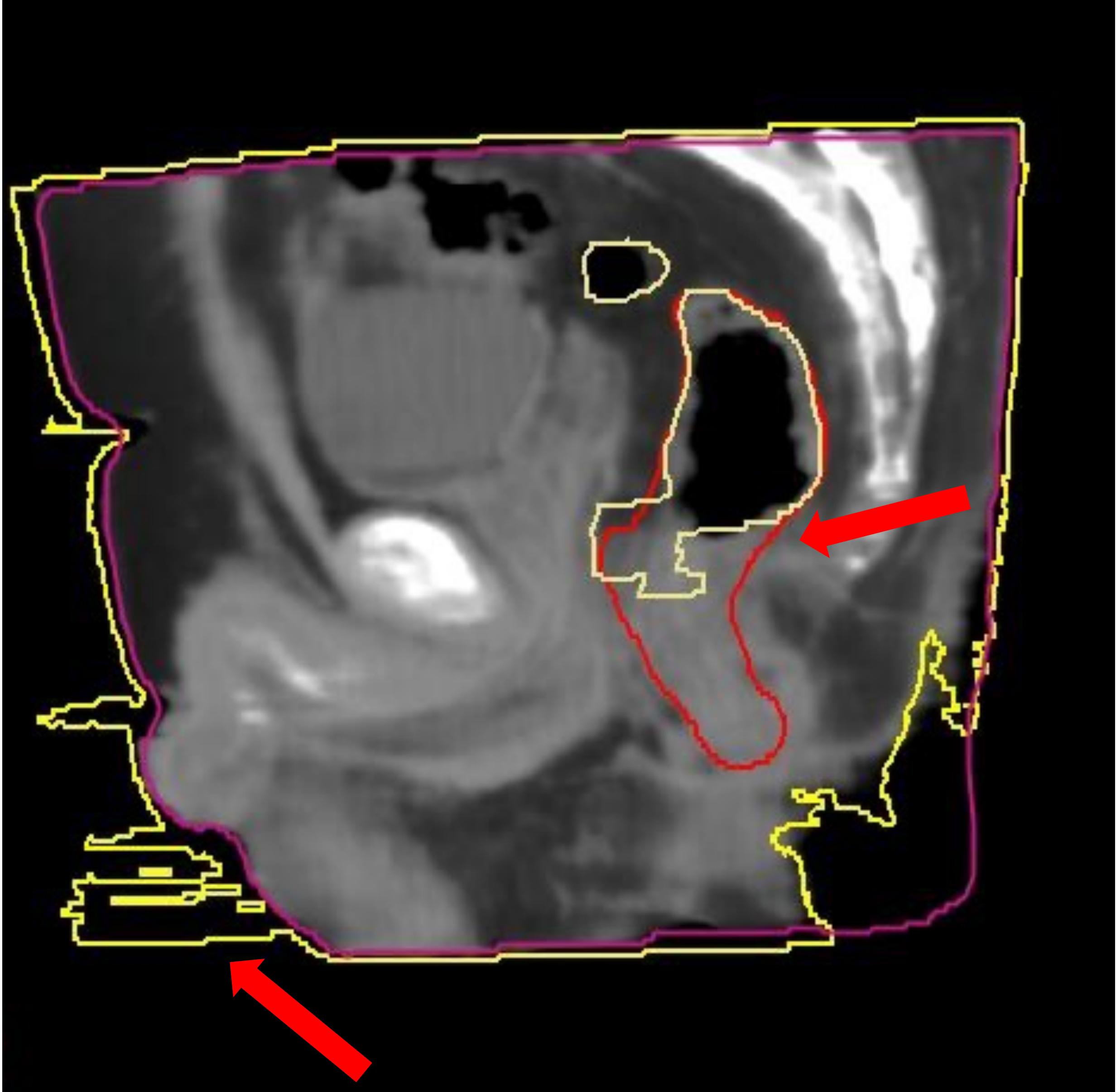

Supplement: Supplementary file 1 [file ACM2-27-e70725-s001.zip › 2026-09415-sup-0011--S.pdf]
