# Supplementary material for: Image‐based evaluation of a commercial AI synthetic CT generator for brain and prostate MR‐only radiotherapy
Source: J Appl Clin Med Phys. 2026 Aug 3;27(8):e70725. doi: 10.1002/acm2.70725 (PMC13433487; doi:10.1002/acm2.70725)
Supplement: Supplementary file 1 [file ACM2-27-e70725-s001.zip › 2026-09415-sup-0012--S.pdf]

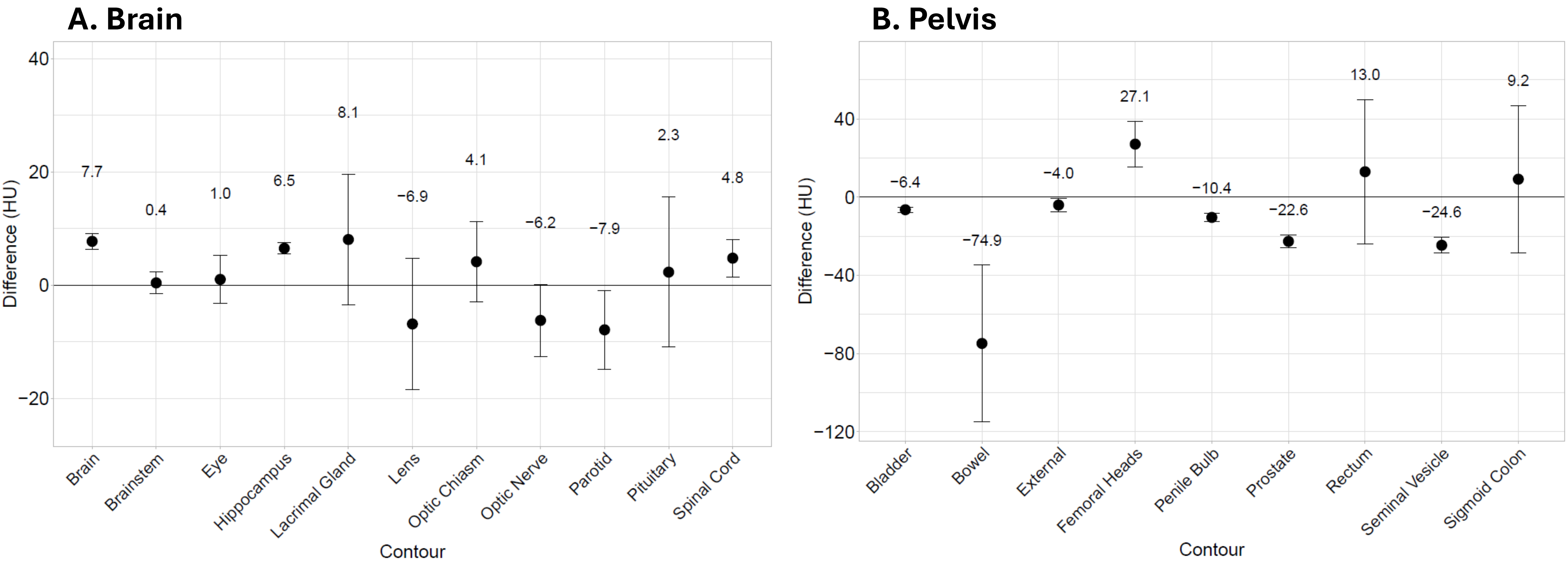
A. Brain
Difference (HU)
40
20
0
−20
7.7
0.4
1.0
6.5
8.1
−6.9
4.1
−6.2
−7.9
2.3
4.8
Brain
Brainstem
Eye
Hippocampus
Lacrimal Gland
Lens
Optic Chiasm
Optic Nerve
Parotid
Pituitary
Spinal Cord
Contour
B. Pelvis
Difference (HU)
40
0
−40
−80
−120
−6.4
−74.9
−4.0
27.1
−10.4
−22.6
13.0
−24.6
9.2
Bladder
Bowel
External
Femoral Heads
Penile Bulb
Prostate
Rectum
Seminal Vesicle
Sigmoid Colon
Contour
