# Supplementary material for: Image‐based evaluation of a commercial AI synthetic CT generator for brain and prostate MR‐only radiotherapy
Source: J Appl Clin Med Phys. 2026 Aug 3;27(8):e70725. doi: 10.1002/acm2.70725 (PMC13433487; doi:10.1002/acm2.70725)
Supplement: Supplementary file 1 [file ACM2-27-e70725-s001.zip › 2026-09415-s01.docx]

Image-based evaluation of a commercial AI synthetic CT generator for brain and prostate MR-only radiotherapy

Morgan Aire^1^, Carson Matthews^2^, Joshua Jones ^1^, David Duong ^1^, Christopher Schneider^1,2^, Sotirios Stathakis^1,2^, Olivia Magneson^1^, Krystal Kirby^1,2^

1. Department of Physics and Astronomy, Louisiana State University, Baton Rouge, LA 70802
2. Department of Radiation Oncology, Mary Bird Perkins Cancer Center, Baton Rouge, LA 70809

Corresponding Author: Morgan Aire

Louisiana State University

Department of Physics and Astronomy

202 Nicholson Hall, Baton Rouge, LA 70803

Tel: (719) 321-0606

Email: [maire1@lsu.edu](mailto:maire1@lsu.edu)

**Submitted:** Journal of Applied Clinical Medical Physics, October 2025

**Resubmitted:** Journal of Applied Clinical Medical Physics, April 2026

**Running Title:** Clinical evaluation of synthetic CT

**Author Contribution Statement:** All authors contributed to the manuscript preparation, and final editing and review.

Data acquisition, analysis: MA, CM, DS, JJ

Data interpretation: MA, CM, DS, JJ, SS, CS, KK, OM

Supervision: SS, CS, KK

Methodology development: MA, CM, KK, SS, CS

**Acknowledgments:** The authors would like to acknowledge Richard Lesieur and Chloe DiTusa for their knowledge and guidance in this subject.

Financial support for this project is provided by the Dr. Charles M. Smith Medical Physics Research Endowment and the Kenneth R. Hogstrom Superior Graduate Student Scholarship in Medical Physics.

**Conflict of Interest Statement:** The authors have no relevant conflicts of interest to disclose.

**Ethical Approval Statement:** Retrospective analysis of patient imaging data was approved by the institutional review board (IRB) under protocol IRBAM-25-0232.
