# Supplementary material for: Image‐based evaluation of a commercial AI synthetic CT generator for brain and prostate MR‐only radiotherapy
Source: J Appl Clin Med Phys. 2026 Aug 3;27(8):e70725. doi: 10.1002/acm2.70725 (PMC13433487; doi:10.1002/acm2.70725)
Supplement: Supplementary file 1 [file ACM2-27-e70725-s001.zip › 2026-09415-sup-0002-SI_Table-S01.docx]

Supplemental Materials

Figure S1. Sagittal slice of a pelvic sCT showing auto-segmentation contour differences for the rectum and external contour. Visible differences in rectum volume are displayed through the TheraPanacea-generated (tan) and CPAI-generated (red) contours. The TheraPanacea-generated external contour (yellow) indicates ghosting from the MR on the anterior side of the patient.

|  | Table S1. Brain Whole-Contour HU Comparison | | | | | | | | | | |
| --- | --- | --- | --- | --- | --- | --- | --- | --- | --- | --- | --- |
| HU Values  (HU ± SE) | | Modality | Contours | | | | | | | | |
|  |  |  | Brain | Bone | Cochleae | Lacrimal Gland | Lens | Optic Chiasm | Optic Nerves | Pituitary | Spinal Cord |
| Minimum | | CT | -441 ± 50 | -3326 ± 698 | 288 ± 53 | -12 ± 6 | 17 ± 4 | -9 ± 2 | -89 ± 16 | -57 ± 26 | -91 ± 25 |
|  |  | CPAI sCT | -326 ± 33 | -293 ± 18 | 833 ± 37 | 25 ± 5 | 43 ± 4 | -70 ± 27 | -56 ± 3 | -179 ± 49 | -120 ± 40 |
|  |  | TP sCT | -350 ± 48 | - | 777 ± 51 | 12 ± 5 | 35 ± 11 | -88 ± 30 | -58 ± 4 | -217 ± 45 | -160 ± 49 |
|  |  | Difference CT - CPAI sCT | -**115 ± 54*** | -**3034 ± 704**** | -**545 ± 62**** | **-37 ± 6**** | **-26 ± 5**** | 61 ± 27 | **-33 ± 17**** | 122 ± 60 | 29 ± 44 |
|  |  | Difference CT - TP sCT | -90 ± 74 | **-** | **-489 ± 78**** | **-25 ± 10*** | **-18 ± 13**** | 79 ± 30 | **-31 ± 16*** | **157 ± 48*** | 69 ± 53 |
| Mean | | CT | 49 ± 3 | 782 ± 29 | 821 ± 59 | 73 ± 4 | 58 ± 4 | 21 ± 2 | 29 ± 2 | 36 ± 3 | 47 ± 4 |
|  |  | CPAI sCT | 59 ± 2 | 646 ± 17 | 1202 ± 53 | 109 ± 10 | 62 ± 2 | 38 ± 5 | 11 ± 7 | 27 ± 12 | 50 ± 2 |
|  |  | TP sCT | 62 ± 1 | - | 1195 ± 54 | 127 ± 13 | 61 ± 2 | 44 ± 6 | 1 ± 8 | 37 ± 10 | 46 ± 3 |
|  |  | Difference CT - CPAI sCT | -10 ± 4 | **135 ± 23**** | **-382 ± 76**** | **-36 ± 12**** | -4 ± 4 | **-17 ± 5**** | **19 ± 7*** | 9 ± 13 | -3 ± 5 |
|  |  | Difference CT - TP sCT | **-14 ± 2**** | **-** | **-374 ± 71**** | **-54 ± 12**** | -3 ± 5 | **-23 ± 5**** | **28 ± 9**** | -1 ± 11 | 1 ± 5 |
| Maximum | | CT | 1389 ± 66 | 17954 ± 3011 | 1690 ± 37 | 306 ± 45 | 87 ± 4 | 65 ± 7 | 361 ± 43 | 109 ± 13 | 220 ± 42 |
|  |  | CPAI sCT | 870 ± 103 | 2627 ± 54 | 1577 ± 75 | 439 ± 46 | 72 ± 2 | 181 ± 37 | 262 ± 13 | 140 ± 17 | 212 ± 22 |
|  |  | TP sCT | 1064 ± 97 | - | 1663 ± 59 | 573 ± 57 | 75 ± 2 | 255 ± 43 | 232 ± 25 | 189 ± 25 | 284 ± 29 |
|  |  | Difference CT - CPAI sCT | **519 ± 118**** | **15327 ± 2995**** | 113 ± 66 | -134 ± 70 | **16 ± 4**** | -116 ± 38 | **100 ± 44*** | -30 ± 24 | 8 ± 53 |
|  |  | Difference CT - TP sCT | **325 ± 108*** | - | 27 ± 50 | **-271 ± 64**** | **12 ± 4*** | **-190 ± 41**** | **129 ± 48*** | **-79 ± 26*** | -65 ± 50 |

Table S1. Brain cross-contour HU comparison between TP- and CPAI-generated contours on the sCT and CPAI contours on the reference CT. Minimum, mean, and maximum HU values are compared with their respective standard error (SE). A positive value indicates a larger reference CT HU value. Bolded values indicate statistical significance using a one-tailed Wilcoxon signed rank test. (** = p < 0.01, * = p < 0.05).

| Table S2. Pelvis Whole-Contour HU Comparison | | | | | | | | | |
| --- | --- | --- | --- | --- | --- | --- | --- | --- | --- |
| HU Values  (HU ± SE) | Modality | Contours | | | | | | | |
|  |  | Bladder | Bone | Femoral Head | Penile Bulb | Prostate | Rectum | Sacrum | Seminal Vesicle |
| Minimum | CT | -125 ± 8 | -127 ± 66 | -109 ± 4 | -59 ± 12 | -229 ± 63 | -806 ± 54 | -254 ± 33 | -109 ± 4 |
|  | CPAI sCT | -88 ± 10 | -12 ± 55 | -70 ± 4 | -8 ± 4 | -24 ± 4 | -405 ± 46 | -75 ± 11 | -55 ± 5 |
|  | TP sCT | -134 ± 57 | - | -293 ± 71 | -10 ± 3 | -38 ± 7 | -405 ± 46 | -253 ± 41 | -75 ± 5 |
|  | Difference CT - CPAI sCT | -**37 ± 12*** | **-114 ± 81*** | **-39 ± 4**** | **-50 ± 12**** | **-204 ± 61**** | **-409 ± 82**** | **-179 ± 32**** | **-55 ± 5**** |
|  | Difference CT - TP sCT | **-3 ± 57*** | **-** | -84 ± 73 | **-49 ± 13**** | **-191 ± 60**** | **-401 ± 78**** | -4 ± 61 | **-37 ± 7**** |
| Mean | CT | 13 ± 1 | 461 ± 11 | 351 ± 10 | 37 ± 1 | 45 ± 2 | -18 ± 19 | 193 ± 9 | 24 ± 1 |
|  | CPAI sCT | 8 ± 1 | 423 ± 10 | 354 ± 9 | 31 ± 2 | 40 ± 3 | -6 ± 7 | 166 ± 6 | 12 ± 2 |
|  | TP sCT | 4 ± 1 | **-** | 387 ± 16 | 29 ± 2 | 39 ± 3 | -20 ± 9 | 136 ± 14 | 10 ± 3 |
|  | Difference CT - CPAI sCT | **5 ± 2*** | **38 ± 8**** | -3 ± 9 | **6 ± 2*** | 5 ± 3 | -4 ± 21 | **27 ± 8**** | 11 ± 3 |
|  | Difference CT - TP sCT | **9 ± 2**** | **-** | **-36 ± 16*** | **8 ± 2**** | 6 ± 4 | **11 ± 21*** | **55 ± 15**** | **15 ± 3**** |
| Maximum | CT | 227 ± 54 | 3733 ± 760 | 1510 ± 10 | 111 ± 4 | 2358 ± 737 | 185 ± 31 | 1276 ± 34 | 200 ± 35 |
|  | CPAI sCT | 116 ± 12 | 1961 ± 23 | 1857 ± 11 | 60 ± 2 | 66 ± 2 | 68 ± 2 | 821 ± 51 | 56 ± 2 |
|  | TP sCT | 115 ± 18 | - | 1920 ± 17 | 61 ± 2 | 66 ± 2 | 66 ± 2 | 793 ± 59 | 56 ± 2 |
|  | Difference CT - CPAI sCT | 112 ± 56 | 1772 ± 763 | **-347 ± 14**** | **51 ± 4**** | **2292 ± 738**** | 118 ± 33 | **455 ± 50**** | **144 ± 34**** |
|  | Difference CT - TP sCT | 99 ± 59 | - | **-410 ± 22**** | **50 ± 5**** | **2291 ± 738**** | **118 ± 31**** | **477 ± 59**** | **146 ± 38**** |

Table S2. Pelvic cross-contour HU comparison between TP- and CPAI-generated contours on the sCT and CPAI contours on the reference CT. Minimum, mean, and maximum HU values are compared with their respective standard error (SE). A positive value indicates a larger reference CT HU value. Bolded values indicate statistical significance using a one-tailed Wilcoxon signed rank test. (** = p < 0.01, * = p < 0.05).

Fig. S2. Deformed mean Hounsfield unit difference between TheraPanacea sCT and reference CT for A. brain and B. pelvic contours. A positive HU difference indicates a greater HU for the sCT. Error bars were derived from standard error of the mean.
