# Supplementary material for: Image‐based evaluation of a commercial AI synthetic CT generator for brain and prostate MR‐only radiotherapy
Source: J Appl Clin Med Phys. 2026 Aug 3;27(8):e70725. doi: 10.1002/acm2.70725 (PMC13433487; doi:10.1002/acm2.70725)
Supplement: Supplementary file 1 [file ACM2-27-e70725-s001.zip › 2026-09415-sup-0005--S.docx]

|  |  | | Table S1. Brain Whole-Contour HU Comparison | | | | | | | |  |
| --- | --- | --- | --- | --- | --- | --- | --- | --- | --- | --- | --- |
| Contour | | Minimum HU (HU ±SE) | | | | Mean HU (HU ±SE) | | | Maximum HU (HU ±SE) | | |
|  |  | CT | | sCT | Difference | CT | sCT | Difference | CT | sCT | Difference |
| Bone | | -2730 ± 987 | | -271 ± 11 | **2459 ± 987**** | 771 ± 32 | 657 ± 21 | **-114 ± 38*** | 17107  ± 4689 | 2653  ± 101 | **-14454**  **± 4690*** |
| Brain | | -412 ± 87 | | -295 ± 33 | 117 ± 93 | 50 ± 2 | 50 ± 1 | 0 ± 2 | 1428 ± 125 | 481 ± 43 | **-947**  **± 132**** |
| Cochlea L | | -144 ± 17 | | -961 ± 92 | **-817 ± 94**** | 644 ± 48 | 1177 ± 108 | 533 ± 118 | 1542 ± 95 | 1424 ± 121 | -114  ± 154 |
| Cochlea R | | -177 ± 14 | | -977 ± 100 | **-800 ± 101**** | 688 ± 20 | 1320 ± 115 | 632 ± 117 | 1640 ± 32 | 1669 ± 128 | 29 ± 132 |
| Lacrimal Gland L | | -9 ± 12 | | 23 ± 4 | **32 ± 13*** | 72 ± 3 | 71 ± 5 | **-1 ± 6**** | 204 ± 37 | 323 ± 67 | **119 ± 77*** |
| Lacrimal Gland R | | -16 ± 11 | | 28 ± 4 | **44 ± 12**** | 68 ± 4 | 82 ± 7 | **14 ± 8*** | 193 ± 35 | 267 ± 25 | 74 ± 43 |
| Lens L | | 12 ± 4 | | 45 ± 2 | **33 ± 6**** | 56 ± 8 | 57 ± 3 | 1 ± 9 | 82 ± 9 | 67 ± 4 | -15 ± 11 |
| Lens R | | 17 ± 3 | | 49 ± 4 | **32 ± 5**** | 63 ± 7 | 57 ± 3 | 6 ± 8 | 90 ± 7 | 72 ± 5 | **-18 ± 9*** |
| Optic Chiasm | | -13 ± 4 | | -4 ± 9 | 9 ± 11 | 18 ± 3 | 23 ± 2 | 5 ± 4 | 56 ± 6 | 49 ± 3 | -7 ± 7 |
| Optic Nerve L | | -143 ± 74 | | -43 ± 7 | **100 ± 74**** | 35 ± 3 | 51 ± 6 | 16 ± 7 | 373 ± 87 | 306 ± 17 | -67 ± 89 |
| Optic Nerve R | | -73 ± 4 | | -58 ± 5 | **15 ± 6*** | 32 ± 3 | 15 ± 5 | -17 ± 6 | 270 ± 41 | 253 ± 23 | -17 ± 47 |
| Pituitary | | -3 ± 5 | | -32 ± 16 | -29 ± 17 | 36 ± 5 | 44 ± 6 | 8 ± 8 | 111 ± 26 | 112 ± 24 | 1 ± 35 |
| Spinal Cord | | -62 ± 18 | | -174 ± 86 | -112 ± 88 | 39 ± 2 | 44 ± 2 | 5 ± 3 | 120 ± 21 | 163 ± 14 | 43 ± 25 |

Table S1. Brain Cross-contour HU comparison between TheraPanacea sCT and reference CT. Minimum, mean, and maximum HU values are compared with their respective standard error (SE). Bolded values indicate statistical significance. (** = p < 0.01, * = p < 0.05).
