# Supplementary material for: Image‐based evaluation of a commercial AI synthetic CT generator for brain and prostate MR‐only radiotherapy
Source: J Appl Clin Med Phys. 2026 Aug 3;27(8):e70725. doi: 10.1002/acm2.70725 (PMC13433487; doi:10.1002/acm2.70725)
Supplement: Supplementary file 1 [file ACM2-27-e70725-s001.zip › 2026-09415-sup-0006--S.docx]

|  |  | | Table S2. Pelvis Whole-Contour HU Comparison | | | | | | | |  |
| --- | --- | --- | --- | --- | --- | --- | --- | --- | --- | --- | --- |
| Contour | | Minimum HU (HU ±SE) | | | | Mean HU (HU ±SE) | | | Maximum HU (HU ±SE) | | |
|  |  | CT | | sCT | Difference | CT | sCT | Difference | CT | sCT | Difference |
| Bladder | | -145 ± 38 | | -87 ± 18 | 58 ± 42 | 14 ± 3 | 8 ± 1 | -6 ± 3 | 203 ± 105 | 176 ± 31 | -27 ± 109 |
| Bone | | -419 ± 107 | | -60 ± 59 | **359 ± 122**** | 485 ± 6 | 454 ± 16 | **-31 ± 17**** | 5295 ± 1289 | 1961 ± 53 | -3334 ± 1290 |
| Femoral Head L | | -97 ± 7 | | -52 ± 9 | **45 ± 11**** | 356 ± 12 | 401 ± 14 | **45 ± 18*** | 1516 ± 19 | 1863 ± 17 | **347 ± 25**** |
| Femoral Head R | | -99 ± 7 | | -77 ± 7 | **22 ± 10*** | 348 ± 15 | 357 ± 14 | 9 ± 21 | 1492 ± 17 | 1857 ± 15 | **365 ± 23**** |
| Penile Bulb | | -35 ± 7 | | -13 ± 7 | 22 ± 10 | 34 ± 2 | 25 ± 3 | **-9 ± 4*** | 101 ± 9 | 53 ± 1 | **-48 ± 9**** |
| Prostate | | -437 ± 128 | | -33 ± 6 | **404 ± 128**** | 43 ± 3 | 29 ± 2 | -14 ± 4 | 4740 ± 1490 | 58 ± 3 | **-4682 ± 1490**** |
| Rectum | | -772 ± 101 | | -526 ± 74 | 246 ± 125 | -23 ± 32 | -18 ± 14 | 5 ± 35 | 176 ± 67 | 63 ± 4 | **-113 ± 67**** |
| Sacrum | | -200 ± 35 | | -70 ± 19 | **130 ± 40**** | 190 ± 13 | 179 ± 13 | -21 ± 18 | 1203 ± 40 | 776 ± 87 | **-427 ± 96**** |
| Seminal Vesicle | | -111 ± 9 | | -69 ± 5 | **42 ± 10**** | 23 ± 2 | 5 ± 3 | **-18 ± 4*** | 116 ± 12 | 49 ± 2 | **-67 ± 12**** |

Table S2. Pelvic Hounsfield unit comparison between CT and sCT for maximum, mean, and minimum HU differences. Bolded values indicate statistical significance. (**= p < 0.01, * = p < 0.05).
